# Color’s Indispensable Role in the Rapid Detection of Food

**DOI:** 10.3389/fpsyg.2021.753654

**Published:** 2021-11-24

**Authors:** Wataru Sato

**Affiliations:** Psychological Process Research Team, Guardian Robot Project, RIKEN, Kyoto, Japan

**Keywords:** color, fast food, food detection, Japanese food, visual search

## Abstract

The detection of food is crucial for our survival and health. Earlier experimental psychological studies have demonstrated that participants detect food more rapidly than non-food stimuli. However, it remains unknown whether color, which was shown to have various influences on food processing, can modulate the detection of food. To address this issue, a psychological experiment was conducted using a visual search paradigm in which photographs of food (fast food and Japanese food) and kitchen utensils were presented alongside images of non-food distractors (cars), with both color and gray images used. Participants used a key to indicate whether one item was different from the rest, and their reaction times (RTs) were measured. RTs for the detection of both food types were shorter than for the kitchen utensils when color images were used, but not when gray images were used; moreover, the RTs were slower for gray images than for color images for both food types but not for kitchen utensils. These results indicate that color facilitates rapid detection of food in the environment.

## Introduction

The detection of food is an initial and crucial stage in the conscious processing of food. For our ancestors, effective food detection supported a higher food intake, permitting the maintenance of energy level and improving the probability of survival. However, because advanced nations are heavily exposed to food and food advertisements, the sensitive detection of food may promote overeating and increase the risk of lifestyle-related diseases in modern life.

Several experimental psychological studies have used visual search paradigms to demonstrate that food items are detected more rapidly than non-food items in the environment (Nummenmaa et al., 2011; de Oca and Black, 2013; Sawada et al., 2017, 2019; Sato et al., 2020). For example, Sawada et al. (2017) investigated the detection of color photographs of fast food, Japanese food, and kitchen utensils among a crowd of non-food distractors (cars). Reaction times (RTs) for the detection of food items were shorter than those for the detection of kitchen utensils, suggesting that food is rapidly detected.

However, whether the color of food items influences detection speed remains unknown. Evolutionary theories have been proposed whereby color vision in primates evolved because of the need to find colored food (Allen, 1879; Polyak, 1957; Regan et al., 2001), suggesting that color processing may affect food detection. Numerous experimental studies have demonstrated that food colors affect various types of processing (e.g., identification) regarding taste and flavor, as well as their hedonic values (for reviews, see Spence et al., 2010; Spence, 2015). For example, Zampini et al. (2007) reported that appropriate coloring of solutions increased correct flavor identification compared with colorless solutions. Hidaka and Shimoda (2014) reported that sweet solutions with appropriate color were rated as sweeter than colorless solutions. One relevant study reported that participants were faster and more accurate at naming colored food images than gray food images (Wurm et al., 1993). The data suggest that color could facilitate rapid processing of food, possibly including food detection. However, because the naming task reflects not only perceptual processing, but also semantic and phonological processing (Humphreys et al., 1999), the effect of color on the detection of food remains to be tested.

**Figure 1:**
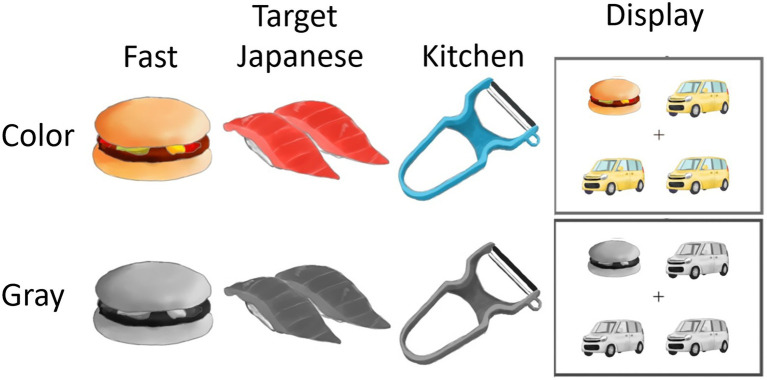
Illustrations of target stimuli (left) and the display (right) in the visual search task in the color and gray modes. Photographic stimuli were used in the actual experiment.

To investigate this issue, a psychological experiment was conducted based on the visual search paradigm in which photographs of food (fast food and Japanese food) and kitchen utensils were presented alongside images of non-food distractors (cars), with both color and gray images used (Figure 1). Images of two food types were used to test the generalizability of the effects. Participants indicated whether an item that differed from the rest was present by pressing keys. RTs were measured and analyzed as the primary dependent variable, as in previous studies (e.g., Sawada et al., 2017). In addition, to evaluate the speed–accuracy trade off, response accuracy and inverse efficiency score (IES; i.e., the combined measure of RT and accuracy; Townsend and Ashby, 1983; Liesefeld and Janczyk, 2019) were also analyzed. Self-reported hunger level was also assessed; its relationship with the color effect on visual search performance was exploratorily analyzed, because some previous studies have reported its effect on visual food processing (e.g., Tapper et al., 2010; Gearhardt et al., 2012; Sawada et al., 2019; however, see Nummenmaa et al., 2011; de Oca and Black, 2013; Sawada et al., 2017). Based on the above-mentioned evidence of the impact of color on food processing, it was predicted that food items would be detected more rapidly than kitchen utensils in color images, but not in gray images.

## Materials and Methods

### Participants

The present study tested 32 young-adult Japanese participants (13 females; mean±*SD* age, 20.6±1.3years). The sample size was determined based on an *a priori* power analysis using G*Power software 3.1.9.2 (Faul et al., 2007). Assuming an *α* level of 0.05, power of 0.80, repeated-measures correlation of 0.5, and medium effect size (*f*=0.25), the results indicated that 28 participants were required for the planned analyses. Participants were recruited *via* advertisements distributed at Kyoto University, and each participant received a pre-paid card for purchasing books worth 1,000 Japanese yen. All participants reported that they had normal or corrected-to-normal visual acuity and no color deficiency. Participants reported their height and weight, and the data showed that their average body mass index was normal (mean±*SD*, 20.8±2.5kg/m^2^; range, 16.6–26.9kg/m^2^). Participants reported their hunger level before the experiment using a five-point Likert scale ranging from 1 (hungry) to 5 (satiated), and the data indicated that most were slightly hungry (mean±*SD*, 2.4±0.9). After the procedures were explained to them, all participants provided written informed consent for participation in this study. This study was approved by the Ethics Committee of the Unit for Advanced Studies of the Human Mind, Kyoto University. The experiment was performed in accordance with the Declaration of Helsinki.

### Experimental Design

A two-factorial within-subject design was used, with the stimulus type (fast food, Japanese food, kitchen utensil) and stimulus mode (color, gray) as factors.

### Apparatus

Stimulus presentation and the recording of responses were controlled on a Windows computer (HP Z200 SFF; Hewlett-Packard Japan, Tokyo, Japan) using Presentation software (Neurobehavioral Systems, Berkeley, CA, United States). Stimuli were displayed on a 19-inch cathode ray tube monitor (HM903D-A; Iiyama, Tokyo, Japan) at a refresh rate of 100Hz and a resolution of 1,024×768pixels. Responses were recorded using a response box (RB-530; Cedrus, San Pedro, CA, United States) at 2–3-ms temporal resolution.

### Stimuli

Four color images of each fast food item, Japanese food item, kitchen utensil, and car were selected from websites as stimuli (Figure 1, left). The target stimuli were cropped and placed on a white background using Photoshop CS6 (Adobe, San Jose, CA, United States). The photographs of fast food (a donut, a hamburger, a piece of fried chicken, and a piece of pizza), Japanese food (a skewer of grilled chicken, Japanese confectionary, noodles, and sushi), and kitchen utensils (a can opener, a frying pan, a kettle, and a peeler) were used as target stimuli. Photographs of cars were used as distractors. These stimuli and the white background have been used in several previous studies; they were reported to reliably promote more rapid detection of food images compared with non-food images in the visual search paradigm (Sawada et al., 2017, 2019; Sato et al., 2020). The mean brightness, contrast, and RGB values of all pixels were matched across the five stimulus types (Sawada et al., 2017). The total caloric content was matched across the two food types (Sawada et al., 2017). One additional item for each stimulus type (fried potatoes, meat and vegetable stew, and scourer) was used only for practice trials. The visual angle of each stimulus was 3.1×3.1°. For gray mode stimuli, all color RGB images were converted from RGB mode to grayscale mode using Photoshop CS6 (Adobe, San Jose, CA, United States).

Four stimuli (either colored or gray) were displayed in a 2×2 array in the visual search task (Figure 1, right). Two sets of stimuli were used: the target-present set, which included one target and three distractors, and the target-absent set, which included four distractors. Identical car images were used as distractors in each trial. Each target stimulus was presented in all four possible locations of the stimulus array.

### Procedure

The experiment was administered individually in a soundproof room. Participants were seated in a comfortable position with a chin rest, which maintained a constant distance of 57cm from the monitor. A total of 240 trials were performed, with an equal number of target-present and target-absent trials. The trials were divided into four blocks of 60 trials each; the stimulus type and stimulus mode conditions were presented randomly within each block. Before the experiment began, participants completed 60 practice trials.

In each trial, a 0.5×0.5° fixation cross was presented for 500ms, followed by a stimulus array, which was displayed until the participant responded. The interstimulus intervals varied from 500 to 800ms. Participants were instructed to indicate whether the photographs in a stimulus array included one anomalous photograph (target present) or were all identical (target absent) by pressing the appropriate key on the keypad with their right or left index finger, as quickly and accurately as possible. They were given no instructions regarding stimulus or food types. The assignment of responsive keys was counterbalanced across participants.

### Data Analysis

The primary dependent variable was the correct-response RTs, as in previous studies that employed visual search tasks involving food items (e.g., Sawada et al., 2017). In addition, the response accuracy (percentage of correct responses) and IES were analyzed. The IES is the most frequently used combined measure of RT and accuracy; it is calculated as the mean correct RTs divided by the proportions of correct responses in each condition for each participant (Liesefeld and Janczyk, 2019). The mean RT/accuracy/IES in target trials was calculated for each condition for each participant, excluding as outliers data with values that were more than ±3 *SD* from the mean for each participant. The RT/accuracy/IES data were subjected to two-factorial repeated-measures ANOVA with stimulus type (fast food, Japanese food, or kitchen utensil) and stimulus mode (color or gray) as factors. Mauchly’s test confirmed that the data satisfied the assumption of sphericity (*p*>0.10). Follow-up simple main effect analyses and multiple comparisons using Ryan’s method (two-tailed) were conducted. When interactions were significant, the main effects were not interpreted due to their problematic properties (Tabachnick and Fidell, 2001). In addition, Pearson’s correlation coefficients were exploratorily calculated between subjective hunger level and the RT/accuracy/IES; difference scores between the color and gray conditions were also calculated and evaluated. All results were considered statistically significant when *p*<0.05. These data analyses were conducted using SPSS 16.0J software (SPSS Japan, Tokyo, Japan).

To test the effect of the individual participant/stimulus on the RT results, an additional analysis was conducted using linear mixed effects modeling. The dependent variables were RTs in single trials; the fixed effects included stimulus type (fast food, Japanese food, or kitchen utensil), stimulus mode (color or gray), and their interaction. Random intercepts for participant and stimulus were added (*cf*. Judd et al., 2012). Model comparison (*cf*. Matuschek et al., 2017) using the Akaike information criterion and Schwarz’s Bayesian information criterion indicated that this model was superior to a model that additionally included random slopes for participant and stimulus. The *F* statistics for the fixed effects were evaluated with the degrees of freedom calculated using Satterthwaite’s approximation. This analysis was conducted using the Statistics and Machine Learning Toolbox of MATLAB 2020a (MathWorks, Natick, MA, United States).

## Results

### Reaction Times

**Figure 2:**
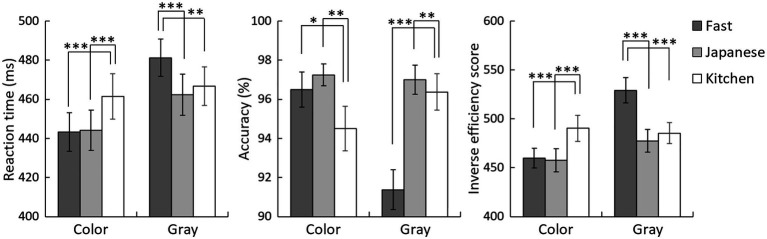
Mean (with *SE*) reaction times (left), percent accuracy (right), and inverse efficiency scores (right) for the detection of fast food, Japanese food, and kitchen utensils in the color and gray modes. Asterisks indicate significant simple effects of stimulus type. ^*^p<0.05; ^**^p<0.01; ^***^p<0.001.

For the RTs in detecting targets (Figure 2, left), the 3 (stimulus type)×2 (stimulus mode) ANOVA revealed significant main effects of stimulus type, *F*(2,62)=4.99, *p*=0.010, $Y\left(t\right)=Ae^{-e^{\beta -kt}}$ = 0.14, and color mode, *F*(1,31)=33.92, *p*<0.001, $\eta_{p}^{2}$ = 0.52, as well as a significant interaction between these factors, *F*(2,62)=13.63, *p*<0.001, $\eta_{p}^{2}$ = 0.31.

Follow-up analyses of the interaction indicated that the simple main effects of stimulus type were significant for both the color and gray modes, *F*(2,124)=8.93 and 8.36, respectively, *p*<0.001 for both. With color images, multiple comparisons indicated that both fast food and Japanese food were detected more rapidly than kitchen utensils, *t*(124)=3.75 and 3.56, respectively, *p*<0.001 for both, whereas no significant difference between fast food and Japanese food was observed, *t*(124)=0.18, *p*=0.855. With gray images, Japanese food and kitchen utensils were detected more rapidly than fast food, *t*(124)=3.91 and 2.99, *p*<0.001 and *p*=0.003, respectively, and no significant difference was observed between Japanese food and kitchen utensils, *t*(124)=0.92, *p*=0.360.

The simple main effects of stimulus mode were significant for fast food and Japanese food, indicating faster detection of food in color images than in gray images, *F*(1,93)=56.34 and 12.88, respectively, *p*<0.001 for both. However, a significant main effect was not observed for kitchen utensils, *F*(1,93)=1.15, *p*=0.294.

To test whether the reported results could be explained by individual participant/stimulus differences, an additional analysis of RTs was conducted using a linear mixed-effects model with the same independent factors as the above ANOVAs, along with random intercepts for participant and stimulus. The results showed that the main effects of stimulus type, *F*(2,11.1)=4.18, *p*=0.044, and color mode, *F*(1,4604.6)=27.10, *p*<0.001, as well as their interaction, *F*(2,4604.7)=6.16, *p*=0.002, were significant. The random intercepts for participant and stimulus showed 95% confidence intervals that did not include zero, (460.37, 768.23) and (14.63, 192.00), respectively. The results indicated that, although RTs differed among participants and stimuli, the interaction between stimulus type and stimulus mode remained significant despite controlling for these effects.

### Accuracy

Regarding the accuracy of target detection (Figure 2, middle), as in the case with the RT analysis, the ANOVA with stimulus type and stimulus mode as factors revealed significant main effects of both stimulus type, *F*(2,62)=11.61, *p*=0.010, $\eta_{p}^{2}$ = 0.27, and stimulus mode, *F*(1,31)=7.79, *p*=0.001, $\eta_{p}^{2}$ = 0.20, as well as a significant interaction between these factors, *F*(2,62)=11.06, *p*<0.001, $\eta_{p}^{2}$ = 0.27.

Follow-up analyses for the interaction revealed that the simple main effect of stimulus type was significant for both color and gray images, *F*(2,124)=4.00 and 18.82, *p*=0.020 and *p*<0.001, respectively. With color images, multiple comparisons showed that both fast food and Japanese food were detected more accurately than kitchen utensils, *t*(124)=1.99 and 2.74, *p*=0.048 and 0.007, respectively, whereas there was no significant difference between the detection of fast food and Japanese food, *t*(124)=0.75, *p*=0.456. With gray images, Japanese food and kitchen utensils were detected more accurately than fast food, *t*(124)=5.60 and 4.98, respectively, *p*<0.001 for both, and no significant difference was observed between Japanese food and kitchen utensils, *t*(124)=0.62, *p*=0.535.

The simple main effects of stimulus mode were significant for fast food, indicating more accurate detection of fast food in color images than in gray images, *F*(1,93)=28.03, *p*<0.001. A non-significant trend for kitchen utensils was observed, indicating more accurate detection of kitchen utensils in gray images than in color images, *F*(1,93)=3.75, *p*=0.056, and no significant effect was observed for Japanese food, *F*(1,93)=0.07, *p*=0.797.

### Inverse Efficiency Score

For IES (Figure 2, right), the ANOVA with stimulus type and stimulus mode as factors again revealed significant main effects of both stimulus type, *F*(2,62)=12.89, *p*<0.001, $\eta_{p}^{2}$ = 0.29, and stimulus mode, *F*(1,31)=45.12, *p*<0.001, $\eta_{p}^{2}$ = 0.59, as well as a significant interaction, *F*(2,62)=23.56, *p*<0.001, $\eta_{p}^{2}$ = 0.43.

Follow-up analyses for the interaction revealed that the simple main effect of stimulus type was significant for both color and gray images, *F*(2,124)=10.91 and 25.43, respectively, *p*<0.001 for both. Multiple comparisons for color images showed that both fast food and Japanese food had lower IES values than did kitchen utensils, *t*(124)=3.88 and 4.20, respectively, *p*<0.001 for both; fast food and Japanese food showed no significant difference, *t*(124)=0.32, *p*=0.750. For gray images, Japanese food and kitchen utensils had lower IES values than did fast food, *t*(124)=5.61 and 6.62, respectively, *p*<0.001 for both; Japanese food and kitchen utensils exhibited no significant difference, *t*(124)=1.02, *p*=0.312.

The simple main effects of stimulus mode were significant for fast food and Japanese food, indicating lower IES values with color than with gray images, *F*(1,93)=82.96 and 6.86, *p*<0.001 and *p*=0.010, respectively. There was no significant effect of kitchen utensils, *F*(1,93)=0.43, *p*=0.514.

### Correlation Between Hunger Level and RT/Accuracy/IES

A correlation coefficient between subjective hunger level and the RT/accuracy/IES under each condition was exploratorily analyzed (Table 1). Difference scores between the color and gray conditions were also calculated and evaluated to test the color effect on visual search performance. No significant correlation was found between subjective hunger level and these visual search performance measures, *r*<0.30, *p*>0.10.

**Table 1:** Pearson’s correlation coefficients between subjective hunger level and visual search performance measures.

Measure	Fast food		Japanese food		Kitchen utensil	
	Color	Gray	Color	Gray	Color	Gray
RT	0.03	0.04	−0.01	0.07	−0.03	0.02
Accuracy	−0.02	−0.10	0.12	0.05	0.08	0.30
IES	−0.07	−0.13	−0.04	0.05	0.02	0.07
RT difference	−0.02		−0.21		−0.09	
Accuracy difference	0.07		0.04		−0.19	
IES difference	0.06		−0.21		−0.08	

*RT=reaction time; IES=inverse efficiency score. Difference scores were calculated between the color and gray conditions. None reached significance (p<0.10)*.

## Discussion

The results in the color mode revealed that RTs for detecting food items, both fast food and Japanese food, were shorter than RTs for detecting kitchen utensils. Accuracy and IES data also showed better scores for both types of food items than for kitchen utensils. These results are consistent with previous findings using only color images (e.g., Sawada et al., 2017) and suggest that the detection of food is more efficient than that the detection of non-food stimuli when the stimuli are presented in color.

More important, the results in the gray mode demonstrated that the gray food images were not detected more rapidly than gray kitchen utensils; in contrast, the gray fast-food images were detected more slowly than the gray kitchen utensil images. The accuracy and IES data showed that these results were not explained by the speed–accuracy trade off, because the accuracy for fast food items was lower than the accuracy for kitchen utensils in the gray mode; IES showed the same patterns with RTs. When RTs were compared between stimulus mode conditions, color items were detected more rapidly than gray items for fast and Japanese food items but not for kitchen utensils. These results are in line with previous findings that the naming of color food images was faster than the naming of gray food images (Wurm et al., 1993) and that food stimuli in the color mode were processed more effectively in terms of sensory and hedonic evaluation compared with the colorless mode (Spence et al., 2010). However, the previous studies did not investigate the effect of color on early food perceptual processing stages. The present study provides the first evidence that color plays an indispensable role in the rapid detection of food.

Unexpectedly, the RT, accuracy, and IES results showed differences between food types such that the influence of colorless presentation of fast food had a greater impact on detection performance than did the colorless presentation of Japanese food. Because there were clear differences in fat contents between these food types, with fast food containing more fat (Sawada et al., 2017), the data suggest that color may be more important for the processing of high-fat food.

Visual search performance of food detection showed no significant correlations with subjective hunger level. These results do not imply that the color effect on food detection could be modulated by hunger status. Several previous studies concerning the detection of food in the visual search paradigm also reported null findings with respect to the modulatory effect of subjective hunger level (Nummenmaa et al., 2011; de Oca and Black, 2013; Sawada et al., 2017; however, see Sawada et al., 2019); thus, the effect of subjective hunger may not be evident in this paradigm. However, our sample size may be insufficient to fully explore this issue, and further investigations are needed.

The current findings showing that color is crucial for the rapid detection of food may have theoretical implications. First, the results provide supportive evidence for the theoretical suggestion that color vision evolved to facilitate food gathering (e.g., Allen, 1879). Several previous animal studies under a naturalistic environment provided supportive empirical evidence for the theory that better color vision facilitates food identification (e.g., Caine and Mundy, 2000; Smith et al., 2003). Anecdotal evidence in humans also suggested that individuals with color deficiency experience difficulty when finding food in a natural environment (Cole, 2004). The current results add empirical evidence in humans to this theory and specify its information-processing mechanism: color vision can facilitate the rapid detection of food. Second, the findings suggest the possibility that the neural mechanisms underlying the rapid detection of food include color processing. This notion is presumably compatible with neuroscientific findings that rapid visual processing of food involves the visual pathway from the pulvinar to the amygdala (Sato et al., 2019); other studies concerning non-food stimuli demonstrated that the pulvinar is involved in binding the shape and color of visual stimuli (Ward et al., 2002), while the amygdala activity changes depending on the level of color harmony (Ikeda et al., 2015). Amygdala involvement may also explain the clear color effect on the detection accuracy for fast food found in the present study, because the amygdala is more strongly involved in the visual processing of high-fat food than low-fat food (Schur et al., 2009). Future neuroimaging studies are warranted to investigate this effect.

The current findings also have practical implications suggesting that color plays an important role in facilitating the perception of food, which could contribute to the consumption of that food. It has been pointed out that many newly created food products containing high fat, sugar, and salt contents have attractive color (Hutchings, 2021). People who hope to control their eating habits and weight should recognize the crucial influence of food color. The present findings imply that a strategy to impair color vision using sunglasses reduces the effective perception of food, which may be helpful for controlling shopping behaviors in food-rich environments, such as supermarkets.

A limitation of this study was that only a few types of food and non-food stimuli were tested, and hence, the generalizability of the findings is limited. Additional research is needed to investigate the effect of color on the detection of various types of stimuli.

In conclusion, this study demonstrated that the detection of fast food and Japanese food was faster than the detection of kitchen utensils when color images were used, but not when gray images were used. Detection was also slower for gray images than for color images for both food types, but not for kitchen utensils. These results indicate that color plays an indispensable role in the rapid detection of food in the environment.

## Data Availability Statement

The original contributions presented in the study are included in the article/Supplementary Material, further inquiries can be directed to the corresponding author.

## Ethics Statement

The studies involving human participants were reviewed and approved by Kyoto University. The patients/participants provided their written informed consent to participate in this study.

## Author Contributions

The author confirms being the sole contributor of this work and has approved it for publication.

## Funding

This study was supported by funds from the Research Complex Program from Japan Science and Technology Agency and Japan Society for the Promotion of Science KAKENHI (18K03174).

## Conflict of Interest

The author declares that the research was conducted in the absence of any commercial or financial relationships that could be construed as a potential conflict of interest.

## Publisher’s Note

All claims expressed in this article are solely those of the authors and do not necessarily represent those of their affiliated organizations, or those of the publisher, the editors and the reviewers. Any product that may be evaluated in this article, or claim that may be made by its manufacturer, is not guaranteed or endorsed by the publisher.

## References

[ref1] Allen G. (1879). The Color-Sense: Its Origin and Development: An Essay in Comparative Psychology. London: Trugner & Co.

[ref2] Caine N. G. Mundy N. I. (2000). Demonstration of a foraging advantage for trichromatic marmosets (Callithrix geoffroyi) dependent on food colour. Proc. Biol. Sci. 267, 439–444. doi: 10.1098/rspb.2000.1019, PMID: 10737399 PMC1690559

[ref3] Cole B. L. (2004). The handicap of abnormal colour vision. Clin. Exp. Optom. 87, 258–275. doi: 10.1111/j.1444-0938.2004.tb05056.x, PMID: 15312030

[ref4] de Oca B. M. Black A. A. (2013). Bullets versus burgers: is it threat or relevance that captures attention? Am. J. Psychol. 126, 287–300. doi: 10.5406/amerjpsyc.126.3.0287, PMID: 24027943

[ref5] Faul F. Erdfelder E. Lang A. G. Buchner A. (2007). G*power 3: A flexible statistical power analysis program for the social, behavioral, and biomedical sciences. Behav. Res. Methods 39, 175–191. doi: 10.3758/BF03193146, PMID: 17695343

[ref6] Gearhardt A. N. Treat T. A. Hollingworth A. Corbin W. R. (2012). The relationship between eating-related individual differences and visual attention to foods high in added fat and sugar. Eat. Behav. 13, 371–374. doi: 10.1016/j.eatbeh.2012.07.004, PMID: 23121790

[ref7] Hidaka S. Shimoda K. (2014). Investigation of the effects of color on judgments of sweetness using a taste adaptation method. Multisens. Res. 27, 189–205. doi: 10.1163/22134808-00002455, PMID: 25577902

[ref8] Humphreys G. W. Price C. J. Riddoch M. J. (1999). From objects to names: a cognitive neuroscience approach. Psychol. Res. 62, 118–130. doi: 10.1007/s004260050046, PMID: 10472198

[ref9] Hutchings J. B. (2021). Evolution and human’s attraction and reaction to colour: food and health. Color. Res. Appl. 46, 140–145. doi: 10.1002/col.22582

[ref10] Ikeda T. Matsuyoshi D. Sawamoto N. Fukuyama H. Osaka N. (2015). Color harmony represented by activity in the medial orbitofrontal cortex and amygdala. Front. Hum. Neurosci. 9:382. doi: 10.3389/fnhum.2015.00382, PMID: 26190992 PMC4486852

[ref11] Judd C. M. Westfall J. Kenny D. A. (2012). Treating stimuli as a random factor in social psychology: A new and comprehensive solution to a pervasive but largely ignored problem. J. Pers. Soc. Psychol. 103, 54–69. doi: 10.1037/a0028347, PMID: 22612667

[ref12] Liesefeld H. R. Janczyk M. (2019). Combining speed and accuracy to control for speed-accuracy trade-offs(?). Behav. Res. Methods 51, 40–60. doi: 10.3758/s13428-018-1076-x, PMID: 30022459

[ref13] Matuschek H. Kliegl R. Vasishth S. Baayen H. Bates D. (2017). Balancing type I error and power in linear mixed models. J. Mem. Lang. 94, 305–315. doi: 10.1016/j.jml.2017.01.001

[ref14] Nummenmaa L. Hietanen J. K. Calvo M. G. Hyönä J. (2011). Food catches the eye but not for everyone: A BMI-contingent attentional bias in rapid detection of nutriments. PLoS One 6:e19215. doi: 10.1371/journal.pone.0019215, PMID: 21603657 PMC3095600

[ref15] Polyak S. L. (1957). The Vertebrate Visual System: Its Origin, Structure, and Function and its Manifestations in Disease With an Analysis of Its Role in the Life of Animals and in the Origin of Man. Chicago: University of Chicago Press.

[ref16] Regan B. C. Julliot C. Simmen B. Viénot F. Charles-Dominique P. Mollon J. D. (2001). Fruits, foliage and the evolution of primate colour vision. Philos. Trans. R. Soc. Lond. Ser. B Biol. Sci. 356, 229–283. doi: 10.1098/rstb.2000.0773, PMID: 11316480 PMC1088428

[ref17] Sato W. Kochiyama T. Minemoto K. Sawada R. Fushiki T. (2019). Amygdala activation during unconscious visual processing of food. Sci. Rep. 9:7277. doi: 10.1038/s41598-019-43733-2, PMID: 31086241 PMC6513994

[ref18] Sato W. Rymarczyk K. Minemoto K. Hyniewska S. (2020). Cultural differences in food detection. Sci. Rep. 10:17285. doi: 10.1038/s41598-020-74388-z, PMID: 33057141 PMC7557965

[ref19] Sawada R. Sato W. Minemoto K. Fushiki T. (2019). Hunger promotes the detection of high-fat food. Appetite 142:104377. doi: 10.1016/j.appet.2019.104377, PMID: 31326438

[ref20] Sawada R. Sato W. Toichi M. Fushiki T. (2017). Fat content modulates rapid detection of food: A visual search study using fast food and Japanese diet. Front. Psychol. 8:1033. doi: 10.3389/fpsyg.2017.01033 28690568 PMC5479904

[ref21] Schur E. A. Kleinhans N. M. Goldberg J. Buchwald D. Schwartz M. W. Maravilla K. (2009). Activation in brain energy regulation and reward centers by food cues varies with choice of visual stimulus. Int. J. Obes. 33, 653–661. doi: 10.1038/ijo.2009.56, PMID: 19365394 PMC2697279

[ref22] Smith A. C. Buchanan-Smith H. M. Surridge A. K. Osorio D. Mundy N. I. (2003). The effect of colour vision status on the detection and selection of fruits by tamarins (Saguinus spp.). J. Exp. Biol. 206, 3159–3165. doi: 10.1242/jeb.00536 12909697

[ref23] Spence C. (2015). On the psychological impact of food colour. Flavour 4:21. doi: 10.1186/s13411-015-0031-3

[ref24] Spence C. Levitan C. A. Shankar M. U. Zampini M. (2010). Does food color influence taste and flavor perception in humans? Chemosens. Percept. 3, 68–84. doi: 10.1007/s12078-010-9067-z

[ref25] Tabachnick B. G. Fidell L. S. (2001). Computer-Assisted Research Design and Analysis. Boston: Allyn and Bacon.

[ref26] Tapper K. Pothos E. M. Lawrence A. D. (2010). Feast your eyes: hunger and trait reward drive predict attentional bias for food cues. Emotion 10, 949–954. doi: 10.1037/a0020305, PMID: 21058840

[ref27] Townsend J. T. Ashby F. G. (1983). Stochastic Modelling of Elementary Psychological Processes. New York: Cambridge University Press.

[ref28] Ward R. Danziger S. Owen V. Rafal R. (2002). Deficits in spatial coding and feature binding following damage to spatiotopic maps in the human pulvinar. Nat. Neurosci. 5, 99–100. doi: 10.1038/nn794, PMID: 11780145

[ref29] Wurm L. H. Legge G. E. Isenberg L. M. Luebker A. (1993). Color improves object recognition in normal and low vision. J. Exp. Psychol. Hum. Percept. Perform. 19, 899–911. doi: 10.1037//0096-1523.19.4.899, PMID: 8409865

[ref30] Zampini M. Sanabria D. Phillips N. Spence C. (2007). The multisensory perception of flavor: assessing the influence of color cues on flavor discrimination responses. Food Qual. Prefer. 18, 975–984. doi: 10.1016/j.foodqual.2007.04.001

